# Surgery or radiotherapy for early-stage cancer study (SORT) target trial protocol: stereotactic ablative radiotherapy (SABR) with curative intent versus surgical resection for early-stage non-small cell lung cancer (NSCLC)

**DOI:** 10.1136/bmjopen-2025-103038

**Published:** 2025-07-13

**Authors:** Eva Kagenaar, David G. Lugo-Palacios, Andrew Hutchings, Ajay Aggarwal, Stephen O’Neill, Bernard Rachet, John Edwards, Corinne Faivre-Finn, Richard Grieve, Surgery or Radiotherapy Study (SORT) group, Ananya Choudhury

**Affiliations:** 1 Department of Health Services Research and Policy, London School of Hygiene and Tropical Medicine Faculty of Public Health and Policy, London, UK; 2 London School of Hygiene and Tropical Medicine, London, UK; 3 Sheffield Teaching Hospitals NHS Foundation Trust, Sheffield, UK; 4 Clinical Oncology Department, The Christie NHS Foundation Trust, Manchester, UK; 5 Division of Cancer Sciences, The University of Manchester, Manchester, UK

**Keywords:** Electronic Health Records, Radiation oncology, HEALTH ECONOMICS, SURGERY, Lung Neoplasms

## Abstract

**Abstract:**

**Introduction:**

Randomised controlled trials have aimed to assess the effectiveness of stereotactic ablative radiotherapy (SABR) with curative intent versus surgical resection for individuals diagnosed with early-stage non-small cell lung cancer (NSCLC) but have failed to recruit sufficient numbers of patients. Non-randomised studies for early-stage NSCLC have reported mixed outcomes following curative SABR versus surgical resection, but did not fully address confounding by indication. The Surgery Or RadioTherapy for early-stage cancer study (SORT) will assess the comparative effectiveness of SABR with curative intent versus surgical resection for NSCLC with a target trial emulation approach, as this can reduce biases in observational studies that aim to estimate the causal effect of interventions.

**Methods and analysis:**

The SORT study will use the National Cancer Registry for individuals diagnosed with early-stage NSCLC in England during 2015–2020 (inclusive) who received SABR with curative intent or surgical resection. These data will be linked to Hospital Episode Statistics, National Radiotherapy Data Set and the Systemic Anti-Cancer Therapy dataset to obtain information on clinical and sociodemographic characteristics and the treatment received. This target trial emulation will define study population eligibility criteria and regimens for SABR with curative intent and surgical resection. We will reduce the risk of residual confounding with instrumental variable analyses that will exploit geographical variation across the National Health Service in England in the use of SABR with curative intent versus surgical resection for early-stage NSCLC. The primary outcome will be 3-year all-cause mortality after treatment initiation. Secondary outcomes will include 3-month, 6-month, 12-month and 24-month all-cause and lung-cancer mortality, time to death, numbers of hospitalisations, incremental costs and incremental cost-effectiveness.

**Ethics and dissemination:**

Ethical approval was obtained from the London School of Hygiene and Tropical Medicine Research Ethics Committee (reference number 29 717–1). Results will be disseminated to clinicians, patients, policy-makers and researchers.

**STRENGTHS AND LIMITATIONS OF THIS STUDY**

- The Surgery Or RadioTherapy (SORT) for early-stage cancer study will use a national cancer registry linked to treatment databases to provide evidence of comparative effectiveness of direct relevance to routine clinical practice.
- The integration of the target trial framework with the instrumental variable analysis will reduce the risk of confounding by indication.
- The national population-based cancer registry has limited detail on tumour location, so the surgical resection group may include patients such as those with centrally located disease, who might not have been eligible for SABR.
- The instrumental variable analysis relies on causal assumptions which can only be partly tested.

## Introduction

 Lung cancer is the leading cause of cancer deaths in England, accounting for 21% of all cancer deaths^1^, and survival is worse than in comparable countries.^2 3^ Non-small cell lung cancer (NSCLC) is the most common type of lung cancer (87.4%).^4^ Surgical resection is the standard of care for early-stage NSCLC with options that include lobectomy, pneumonectomy, bilobectomy or wedge resection.^5^ The National Institute for Health and Care Excellence (NICE) recommends stereotactic ablative radiotherapy (SABR)^5^ as an alternative to resection, recognising that SABR is well-tolerated and has better survival rates than conventional radical radiotherapy.^6^,^8^ NICE guidelines highlight the need for research comparing SABR to surgery, given that SABR is a non-invasive, organ-preserving treatment. If SABR is as effective as surgery, then it could be a preferable option for some people with NSCLC,^5^ while also helping to alleviate pressure on limited surgical resources and reduce waiting times. However, NICE guidelines also highlight the absence of unequivocal evidence on comparative effectiveness from randomised controlled trials (RCTs) comparing SABR vs surgical resection for early-stage NSCLC.

Several RCTs comparing SABR versus surgical resection for early-stage NSCLC, including ROSEL,^9^ STARS,^10^ SABRTooth^11^ and ACOSOG 4099/RTOG 1021,^12^ have closed prematurely because of slow accrual. This challenge is largely driven by patient and clinician preference for either treatment modality.^13^ A pooled analysis of the ROSEL and STARS trials suggested that SABR and surgical resection may result in similar 3-year overall and recurrence-free survival.^14^ However, the limited sample size and short follow-up meant that the findings were highly uncertain and difficult to interpret for clinical practice. Currently, two ongoing phase III RCTs, VALOR^15^ (accrual ongoing) and STABLEMATES^16^ (accrual completed), are investigating the comparative outcomes of SABR and surgical resection for early-stage NSCLC. Pending the results of these RCTs, clinical decision-makers and patients lack high-quality evidence to guide treatment decisions.

While SABR is typically recommended for individuals who are ineligible or who decline surgical resection, growing observational evidence suggests that, for some patients, SABR may offer similar outcomes to surgical resection.^17^ Several single-centre^18^,^21^ and multicentre^22 23^ non-randomised studies suggest that overall survival between 1 year and 5 years after diagnosis may be similar among individuals who receive SABR or surgical resection.^18 20 22^ One single-centre study reported higher cancer-specific survival, but lower overall survival following SABR versus surgical resection.^21^ Some meta-analyses of non-randomised studies have found similar lung-cancer-specific survival at 5 years,^24^ others that overall mortality is similar at 1–5 years after diagnosis,^25 26^ while a third group has reported that overall survival at 5 years is lower following SABR compared with lobectomy or sublobar resection.^24 27^ A major concern is that none of these studies have attempted to reduce the risk of unmeasured confounding, which may have led to biased estimates of comparative effectiveness and limited the studies’ relevance for clinical decision-making.

Evidence about the cost-effectiveness of SABR versus surgical resection for early-stage NSCLC is also limited. A recent systematic review by Maia *et al*^28^ identified six studies that compared the direct healthcare costs of SABR versus surgery. Two of the studies concluded that SABR was cost-effective compared with surgical resection,^29 30^ and the study by Puri *et al*^31^ found that surgery was more cost-effective than SABR, while other studies reported mixed results depending on the surgical procedures compared.^32^,^34^ The variation in results from these studies is likely driven by differences in study design, the surgical procedures compared, and the costs considered.^28^

This uncertainty in the evidence about the effectiveness and cost-effectiveness of SABR with curative intent versus surgical resection has contributed to variation across National Health Service (NHS) trusts in England in the proportion who received SABR for early-stage NSCLC.^4 35^ These variations may partly reflect differences in the preferences of thoracic oncology teams, the number of SABR centres which increased from approximately 20 (2015) to 37 (2019),^36^ and in the fitness and demographic of patients diagnosed with early-stage NSCLC.^17^ Older individuals, who represent the fastest growing subpopulation presenting with early-stage NSCLC,^37^ are more likely to have multiple long-term conditions and higher mortality rates,^38^ making them less suitable for surgical resection and more likely to receive SABR.^17^ During the COVID-19 pandemic, there was reduced surgical capacity, and so the uptake of radiotherapy, including SABR, increased in many centres.^39^,^41^

To address the challenges faced by previous non-randomised studies that have compared SABR to surgical resection for NSCLC, this study will leverage a national-level linked dataset and integrate a target trial emulation design with an instrumental variable analysis. The study will be designed to exploit the variations in use of SABR with curative intent across NHS trusts and over time to enable us to compare SABR with curative intent to surgical resection for similar patients who would be eligible for either modality. This approach aims to provide an accurate assessment of the comparative effectiveness and cost-effectiveness of SABR versus surgical resection for early-stage NSCLC.

### Aims and objectives

This study aims to evaluate the comparative effectiveness and cost-effectiveness of SABR with curative intent versus surgical resection for early-stage NSCLC, using cancer registry data from the National Disease Registration Service (NDRS) in England.^42^

The study objectives are:

1. To estimate the comparative effectiveness of SABR with curative intent versus surgical resection for the overall population and subgroups with early-stage (tumour: 1-2, node: 0, metastases: 0 (T1-2N0M0)) NSCLC.
2. To evaluate the comparative cost-effectiveness of SABR with curative intent versus surgical resection for the overall population and subgroups with early-stage (tumour: 1-2, node: 0, metastases: 0 (T1-2N0M0)) NSCLC.

## Methods and analysis

### Overview

The SORT study will use large-scale linked national cancer registry data for England to evaluate the comparative effectiveness and cost-effectiveness of SABR with curative intent versus surgical resection for early-stage NSCLC over a 3-year follow-up period. We will integrate an emulated target trial design with an instrumental variable analysis to reduce the risk of confounding.^43^,^46^ Target trial emulation applies the design principles of RCTs to observational data in defining a hypothetical RCT (the emulated target trial) and can mitigate common design biases inherent in observational studies.^47^ In particular, our target trial will predefine patient eligibility, treatment strategies and outcomes. In stipulating these definitions, this protocol is informed by published and ongoing RCTs and non-randomised studies of SABR versus surgical resection for early-stage NSCLC, pilot data from the national cancer registry, and expert opinion from a clinical panel. The SORT NSCLC clinical panel comprised nine cardiothoracic surgeons and radiation oncologists who met online in April 2024 to inform the study protocol. Input from this panel focused on refining eligibility criteria, treatment definitions and anticipated effect sizes. The accompanying cost-effectiveness analysis (CEA) will assess whether SABR with curative intent versus surgical resection is cost-effective overall and for specific patient subgroups.

### Data

An overview of the datasets and their purpose is presented in table 1. We will use England’s NDRS Cancer Registry data to identify individuals aged 18 and over diagnosed with early-stage (T1-2N0M0) NSCLC in 2015–2020 inclusive. The Cancer Registry collects comprehensive data on all malignant tumours diagnosed in England recorded by NHS healthcare providers as part of routine care. These data include information on tumour and patient characteristics, geographical location, vital status (alive, deceased, emigrated or lost to follow-up), and for this study, it will use follow-up data until the end date of 31 December 2023.

**Table 1:** Overview of the different datasets within the ‘NDRS linked data’ and their proposed use in the SORT target trial for NSCLC

Dataset	Purpose in the study
National Disease Registration Service (NDRS) Cancer Registry	Identify individuals diagnosed with T1-2N0M0 NSCLC between 2015 and 2020 (base case) and between 2015 and 2023 (alternative analyses) Obtain vital status Collect patient and tumour characteristics (e.g., age at diagnosis, income deprivation, sex, tumour stage, histology)
Hospital Episodes Statistics inpatient and outpatient	Identify individuals who underwent surgical resection within 6 months of diagnosis Collate resource use (e.g., hospitalisations, outpatient visits, surgical procedures) Collect measured confounders (e.g., comorbidities and frailty) Define time zero (date of curative surgery)
Radiotherapy Data Set	Identify individuals who received SABR within 6 months of diagnosis (base case) Collate use of different radiotherapy regimens Identify time zero (date of first dose or radiotherapy)
Systemic anti-cancer therapy	Collate use of different immuno-oncology and chemotherapy regimens including those with palliative intent
Cancer outcomes and services data set	Obtain Zubrod performance status

M, Metastases; N, node; NSCLC, non-small cell lung cancer; T, tumour.

NDRS Cancer Registry data is linked to the inpatient and outpatient Hospital Episodes Statistics (HES) for all patients treated by the NHS in England. HES data contain clinical information, such as diagnoses, medical procedures, patient’s sociodemographic characteristics and geographical information for all outpatient and inpatient visits in England. The registry is also linked to the Radiotherapy Data Set and Systemic Anti Cancer Therapy data which contain records of all radiotherapy and chemotherapy provided by the NHS (See table 1). From the NDRS Cancer Registry linked dataset (the ‘NDRS linked data’), we will obtain required information on patient, clinical and contextual measures, treatments received and outcomes. A detailed description of the NDRS linked data completeness is provided elsewhere.^48^

### Target trial design

We will emulate a target trial by conceptualising this non-randomised study as if it were an RCT in clearly defining the eligibility criteria, study population and treatment regimens (table 2).^43 47^ These definitions are informed by ongoing and prematurely closed RCTs comparing the effectiveness of SABR versus surgical resection for early-stage NSCLC.^9 10 15 16 49^ Even after applying the target trial eligibility criteria, it is likely that unobserved confounders (e.g., tumour location) will remain imbalanced between the comparison groups. We will use an instrumental variable (IV) analysis to reduce the risk of bias due to unobserved confounding^44 46 50^ (see the Analyses section for details).

**Table 2:** Overview of the emulated target trial components and their definitions

Protocol component	Emulation protocol
Inclusion criteria	People with first primary NSCLC diagnosis (T1 – T2 N0 M0) between 1 January 2015 and 31 December 2020, according to ICD-10 C34 (all ICD-O-3 morphology codes except those between 8041 and 8045) who received SABR with curative intent or underwent surgical resection within 30 days before or 6 months after diagnosis (base case)
	Aged 18–79 years old if Zubrod performance status 0–2, age 80+if Zubrod performance status 0–1
Exclusion criteria	Synchronous lung cancerPregnancy at time of diagnosis
	Previous thoracic radiotherapy within 5 years prior to diagnosisPrevious metastatic malignancy within 5 years prior to diagnosis
	No surgery or radiotherapyOther types of radiotherapy as index treatment
Treatment strategies	Radiotherapy with curative intent: Stereotactic Ablative Radiotherapy (SABR)Surgical resection: Video-assisted or open thoracic lobectomy+/-mediastinal lymph node dissection (MLND); segmentectomy+/-MLND; wedge resection+/-MLND
Assignment procedure	Two approaches to address confounding: Randomisation will be emulated with an instrumental variable analysis that aims to balance observed and unobserved baseline prognostic measures between the comparison groups (base case analysis). The proposed instrument is the proportion of eligible patients treated with SABR versus surgical resection within the cancer network Randomisation will be emulated via a double robust method- inverse probability of treatment weighting with regression adjustment (alternative analysis)
Time zero	Date of treatment start
Follow-up	Follow-up begins on date of treatment assignment (time zero) and ends 3 years after baseline (base case)
Outcome	Primary outcome: all-cause mortality at 3 years from the date of treatment receipt
	Secondary outcomes: all-cause and lung-cancer mortality at 3 months, 6 months, 12 months, 24 months, and 36 months; time to death; number of days in hospital in 12 months after treatment start; incremental costs; and incremental cost-effectiveness (incremental net health benefits).
Causal contrast of interest	Intention-to-treat effect (patients analysed according to their allocated treatment, regardless of whether they did not fully adhere to treatment or switched to other treatments)

NDRS, National Disease Registration Service; NSCLC, non-small cell lung cancer.

#### Eligibility criteria

**Figure 1:**
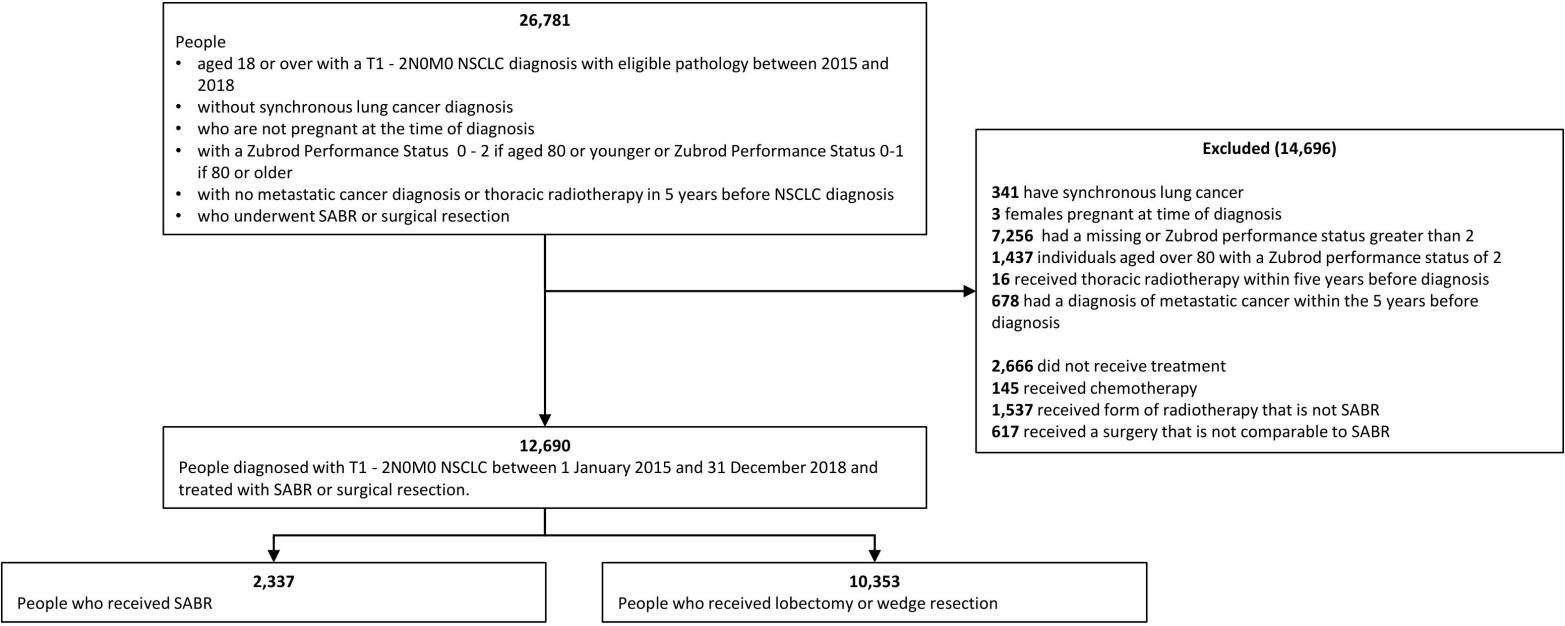
Flow diagram illustrating the identification of individuals who received SABR with curative intent or surgical resection for T1-2N0M0 NSCLC diagnosed between 1 January 2015 and 31 December 2018 - pilot NRDS data. NSCLC, non-small cell lung cancer; SABR, stereotactic ablative radiotherapy; TNM, tumour, node, metastases.

A flowchart demonstrating the application of the eligibility criteria to a pilot version of the ‘NDRS linked data’ which included people diagnosed during 2015–2018 is presented in figure 1. The study will include individuals aged 18–79 years old with a recorded Zubrod performance status of 0–2^51^ and those aged 80 years or above with a performance status of 0–1, who were diagnosed with early-stage (T1-2N0M0) NSCLC during 2015–2020 and underwent SABR or surgical resection less than 30 days before or 6 months after diagnosis. We will exclude individuals who had a pregnancy-related HES inpatient or outpatient visit in the 9 months following diagnosis and those who had either synchronous lung cancer or a diagnosis of metastatic cancer in the 5 years before their NSCLC diagnosis or thoracic radiotherapy in the 5 years before their NSCLC diagnosis. We will also consider alternative eligibility criteria as part of the alternative analyses.

#### Covariates and time zero

The target trial approach requires baseline covariates to reduce the risk of confounding and to undertake subgroup analyses. From the NDRS Cancer Registry, we will obtain demographic and tumour data including: age at diagnosis, sex, year of diagnosis, tumour histology (adenocarcinoma, squamous, other), tumour stage (T1N0M0, T2N0M0), ethnicity (white, black, South-Asian, other, missing) and income quintile of the Index of Multiple Deprivation.^52^ Zubrod performance status^51^ will be obtained from the Cancer Outcomes and Services Data. From the HES data, we will define comorbidities from inpatient and outpatient visits that include the following diagnoses in the 5 years before the NSCLC diagnosis: myocardial infarction, stroke, heart failure, chronic obstructive pulmonary disease, peripheral vascular disease, diabetes, and interstitial lung disease.

We will define ‘time zero’, which is analogous to the time of randomisation as it is when eligibility criteria are met and follow-up starts, to be the first date that SABR with curative intent is received or surgical resection is undertaken. We will define covariates for ‘seasonality’ by the month either treatment modality was first received, and ‘treatment waiting time’ as the time between the decision to treat and ‘time zero’. We will capture quality of care measures including waiting times and the volume of surgical resections performed in the 12 months before time zero. We will define the cancer alliance and the NHS region within which the surgical hub is located, to account for regional variations.

#### Treatment strategies

Table 3 shows the SABR regimes considered in this study which follow the Royal College of Radiologists’ guidelines,^6^ practice during the COVID-19 pandemic^41^ and expert inputs from the study’s NSCLC clinical panel. Table 3 also presents the codes used to define surgical resection with curative intent for NSCLC. For both treatment modalities, we will apply a grace period of 30 days before and 6 months after NSCLC diagnosis to account for the prioritisation of diagnosis by pathology as defined by the European Network of Cancer Registries^53^ and to allow for treatment waiting times.

**Table 3:** Overview of the regimens and procedures included within the definition of curative SABR and surgical resection

Treatment definition	Source
54 Gy in 3 fractions over 5–8 days	Clinical Oncology Radiotherapy dose fractionation, Royal College of Radiologists^1^
55 Gy in 5 fractions over 10–14 days	Clinical Oncology Radiotherapy dose fractionation, Royal College of Radiologists^1^
60 Gy in 5 fractions over 10–14 days	Clinical Oncology Radiotherapy dose fractionation, Royal College of Radiologists^2^
60 Gy in 8 fractions over 10–20 days	Clinical Oncology Radiotherapy dose fractionation, Royal College of Radiologists^1^
30–34 Gy in 1 fraction	Faivre-Finn *et al*^3^
60–85 Gy in 5 fractions	Input from clinical panel
50 Gy in 5 fractions	Input from clinical panel
50 Gy in 8 fractions	Input from clinical panel
OPCS-4: E54.3 lobectomy	Input from clinical panel
OPCS-4: E54.4 excision of segment of lung	Input from clinical panel
OPCS-4: E54.5 partial lobectomy of lung NEC	Input from clinical panel

Gy, Gray; NEC, not elsewhere classified; OPCS-4, Office of Population Censuses and Surveys Classification of Interventions and Procedures, version 4; SABR, stereotactic ablative radiotherapy.

#### Follow-up period and outcomes

The primary outcome is all-cause mortality 3 years from the start of treatment (time zero). We will also report all-cause and lung-cancer specific mortality at 3 months, 6 months, 12 months, 24 months and 36 months as secondary outcomes. We will define lung-cancer attributable mortality as those deaths which include lung cancer (International Classification of Diseases-10: C34) as a cause of death. Multivariable flexible hazard models^54^,^56^ will be fitted to estimate the effect of SABR versus surgical resection on time to death for both all-cause and lung-cancer specific mortality. For these time-to-death analyses, individuals treated during 2015–2023 inclusive will be included to maximise follow-up duration.

The number of days in hospital in the 12 months after the start of treatment will be recorded. We will use information on reasons and route of admission (eg, via emergency room) in the 90 days following the start of treatment to identify admissions attributable to grade three or four adverse events following either treatment modality (see also CEA).

In the pilot NDRS linked registry data, information was available for the primary endpoint (3-year mortality) for each individual in the eligible population. Covariate information was also complete for all the required covariates for the eligible population with the exception of ethnicity, for which the proportions with missing data were low and similar between the comparison groups (1.71% for SABR and 1.34% for surgical resection). The analysis will therefore use observations with complete case information which assumes the missing ethnicity data are independent of the outcome given the covariates included in the analytical models.^57^

#### Causal contrast of interest

The analysis will follow an intention-to-treat approach with individuals included in the analysis according to whether they received SABR or had surgical resection at time zero, irrespective of the treatments they received subsequently. Individuals will therefore continue to contribute to the analysis until they are censored by death or the end of the 3-year follow-up period (base case) or 31 December 2023 (alternative analysis).

#### Sample size calculations

For the sample size calculations, a between-group difference in 3-year all-cause mortality of 7.5% (absolute risk scale) was defined as of clinical importance drawing on precedent observational studies and RCTs, and informed by the clinical panel. From the pilot linked registry data, the 3-year mortality rate for the eligible population who had surgical resection was 19.8%. We followed methodological recommendations for sample size calculations with IV analyses and present the required sample size under different assumptions about the proportions predicted by the IV to receive SABR versus surgery (the compliance rate).^58^ Table 4 shows that, with an assumed IV compliance rate of 0.7, the sample size that would be required to achieve power of 80% at the 5% (two-sided) level of statistical significance is 2,887 of whom at least 520 would need to be the SABR group. Based on the pilot data which included diagnoses and treatments in 2015–2018, we anticipate that in the final analysis sample for years 2015–2020, there will be at least 18 000 (3000 SABR and 15 000 surgical resection).

**Table 4:** Required sample size (N) for SABR and surgical resection according to magnitude of effect size (absolute differences: SABR versus surgical resection) on 3-year mortality at 80% power, 5% level of statistical significance and assuming a ‘moderate’ level of instrument strength, corresponding to a compliance rate of 0.7

Effect size	Surgery	SABR	Total
−7.5%	2625	576	3201
−5.0%	5830	1280	7110
+5.0%	5443	1195	6638
+7.5%	2367	520	2887

SABR, stereotactic ablative radiotherapy.

### Cost-effectiveness analysis

#### Overview

We will assess the comparative cost-effectiveness of SABR versus surgical resection for individuals with early-stage NSCLC. The CEA will take a hospital perspective and report costs, outcomes and cost-effectiveness over a 3-year time horizon. This perspective and time horizon are anticipated to be sufficient to capture the important differences in mean costs and mean outcomes between the comparison groups. The CEA will incorporate individual-level resource use and mortality data from the linked NDRS data, combined with unit costs and health-related quality of life (HRQoL) estimates from the literature.^59^,^61^ The CEA will follow the assessment of comparative effectiveness in using IV methods to reduce the risk of residual confounding. We will report the net health benefits^62^ of SABR with curative intent versus surgical resection overall and for the subgroups of interest described in the Main analyses section.

#### Resource use and unit costs

From the linked NDRS data, we will identify resource use for those categories anticipated to drive incremental costs, including the delivery of SABR, surgical procedures, hospital inpatient stays (including all readmissions, subsequent surgery, palliative care), outpatient visits, diagnostic procedures and subsequent treatment (eg, systemic therapies, radiotherapy or salvage surgeries). We will extract data on the receipt of SABR as well as the Office of Population Censuses and Surveys Classification of Interventions and Procedures codes of all surgical resections and operative procedures performed on each eligible patient. For hospital inpatient stays, we will distinguish between the time spent in critical care and on general wards.

Unit costs, including those for SABR and surgical strategies, will be taken from the NHS Cost Collection^63^ and the Personal Social Services Research Unit Cost databases.^64^ We will combine resource use with unit costs to report total costs per patient over 3 years (see also the Alternative analyses section).

#### Outcomes for the CEA

We will calculate the number of life years from the date of treatment start up to 3 years (base case) and for the maximum observation period available (alternative analyses). The NRDS linked data will be used to identify cancer recurrence and disease progression based on diagnoses and procedures captured in subsequent hospital admissions, registration of new tumours and treatment receipt, including surgery, chemo-oncology, immuno-oncology therapy and radiotherapy using a novel approach developed for bowel cancer.^65^ The IV analysis will compare the adjusted proportions of cancer recurrences, common severe adverse events (eg, pneumonia, respiratory distress syndrome or failure and myocardial infarction)^66^ following SABR versus surgical resection. Pending these results, we will apply appropriate HRQoL estimates from the literature.^59^,^61^ For individuals who died of lung cancer, lower HRQoL will be assigned in the six months prior to death.

We will combine survival time with appropriate HRQoL estimates from the literature and use the ‘area under the curve’ approach^67^ to report quality adjusted life years (QALYs) at 3 years from treatment start. We will calculate the incremental net benefit by valuing the incremental QALYs (difference in mean QALYs for SABR vs surgical resection) with alternative threshold levels for cost per QALY gain, including those specified by NICE (eg, £20 000 and £30 000).

### Planned statistical analyses

#### Main analyses (base case)

**Figure 2:**
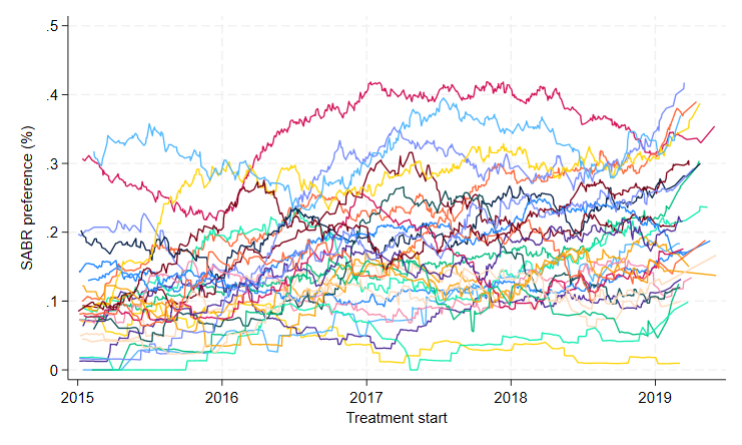
The proportion of eligible patients in each cancer referral network who had SABR with curative intent versus surgery in the NDRS pilot data (2015–2018). NDRS, National Disease Registration Service; SABR, stereotactic ablative radiotherapy.

For the main analysis (base case) we will use an IV to reduce the risk of confounding that is due to unmeasured baseline variables, such as tumour location, as well as measured confounders, such as age or cancer stage.^58^ The IV will exploit natural variation across the cancer networks and over time in the proportion of eligible individuals who had SABR rather than surgical resection (see figure 2). This natural variation implies that people with a similar prognosis at baseline will differ in whether they receive SABR or have surgical resection simply according to the cancer network or the time period in which they have treatment. The definition of cancer referral networks aligns with the ‘hub’ and ‘spoke’ model of care for lung cancer^68^ and comprises a surgical centre (the hub) with a multidisciplinary team who inform the decision of SABR versus surgical resection and the surrounding hospitals (the spokes) that refer individuals with NSCLC to the centre. The IV is the historic proportion of eligible individuals with NSCLC within each cancer referral network who had SABR versus surgical resection. This proportion is calculated for each individual for the six months before that individual starts either treatment.

**Figure 3:**
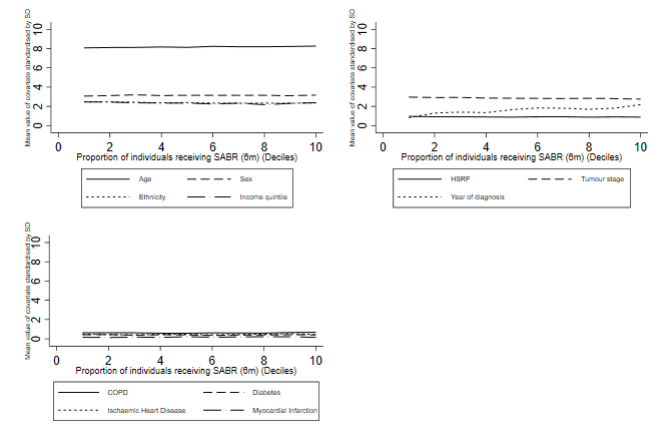
Balance of baseline covariates across different levels of the instrumental variable, the proportion within the cancer referral network who received SABR with curative intent versus surgery. 6m, six months prior ;COPD, chronic obstructive pulmonary disorder; HFRS, Hospital Frailty Risk Score; SABR, stereotactic ablative radiotherapy.

A valid instrument must meet three main conditions.^58^ First, the instrument must predict the treatment received, which can be formally assessed.^69 70^ For the instrument to be of sufficient strength, the F-statistic summarising the association between the IV and the treatment received must exceed 100.^71^ In the NDRS pilot data, the corresponding F-statistic^70^ was 329. Second, the instrument must be independent of baseline covariates which are prognostic of the outcome of interest. This can be evaluated for the observed baseline measures. We found that the observed baseline variables were balanced across different levels of the instrument (figure 3). Third, the instrument must only have an effect on the outcomes through the treatment received, which cannot be evaluated empirically. If there were imbalances in measured covariates across levels of the IV this would raise concerns about the second and third IV assumptions. We will address this potential risk of confounding by adjusting for any residual differences in measured contextual and temporal confounders in the second stage (outcome) regression. These differences may pertain to variations in the quality of cancer care provided across the networks. By adjusting for these variables, we can make a weaker assumption that the IV, the proportion in the network who received SABR, does not have a direct effect on the outcome *after* adjusting for any differences in contextual variables pertaining to the quality of care and the time period.

The IV approach will report comparative effectiveness and cost-effectiveness across the overall eligible study population. The first stage models will estimate the probability of receiving SABR versus surgical resection given baseline covariates and the IV.^72^ The second stage outcome models will use the general linear models framework and choose the specific model according to whether the endpoint is binary (e.g., mortality at 3 years), continuous (e.g., costs) or time-to-event (e.g., time to death). All estimates will be reported with bootstrapped CIs that make appropriate allowance for clustering.

The IV approach will also report comparative effectiveness and cost-effectiveness according to prespecified subgroups including sex; age group; pre-existing cardiovascular disease or not; hospital frailty score (corresponding to fit, low or intermediate risk); Zubrod performance status (0–2), tumour stage, tumour histology and year of diagnosis.

#### Alternative analyses

We will conduct alternative analyses under four broad categories to check the robustness of the base case results. The first set of alternative analyses will pertain to the inclusion criteria to examine whether the results are robust to alternative inclusion criteria. For example, we will consider the combination of age and performance status suggested in the SABRTooth feasibility study protocol^11^ or performance status 0–1 as in VALOR^15^ to identify patients suitable for a trial comparing SABR versus surgical resection. We will also look at the impact of excluding observations who did not have either treatment within three (vs six months) of diagnosis. Both alternative inclusion criteria reflect an alternative definition of the population for whom there may be more equipoise between SABR and surgical resection. Second, we will consider the impact of COVID-19, for example by excluding individuals treated during the first wave of COVID-19 (1 February–30 June 2020). Third, we will consider analysis methods that make different causal assumptions to the IV analysis. For example, we will apply double-robust methods, such as inverse probability weighting with regression adjustment, which assumes no unobserved confounders.^73^

Fourth, for the CEA, we will consider alternative analyses that examine the impact of using the maximum available survival data in extrapolations to time horizons of 5 years, 10 years and the lifetime.^74^ We will also consider alternative sources for the requisite HRQoL data and alternative criteria for defining NSCLC recurrence.

### Strengths and limitations

This study will assess the comparative effectiveness and cost-effectiveness of SABR versus surgical resection for early-stage NSCLC. By using national cancer registry data, this study identifies a large and diverse population that is directly relevant for clinical decision making. A key strength of this study is its ability to report results stratified by clinically relevant risk factors. Combining the target trial design with the IV analysis can help reduce bias from confounding by indication, enhancing the robustness of our findings.

The study protocol will have a similar design to the relevant RCTs, for example, ACOSOG 4099/RTOG 1021, STARS, ROSEL, VALOR, STABLEMATES, but also allow for appropriate representation of those subgroups underrepresented in trials. To enhance the clinical relevance and validity of the study, a clinical panel informed aspects of the study, including the eligibility criteria, treatment definitions, outcome measures and expected effect sizes.

A limitation of the study is that the national population-based cancer registry has limited detail on tumour location, so some individuals included, such as those with centrally located disease who undergo surgical resection, might not have been eligible for SABR. The linked NDRS registry data does not capture information on all outcomes of interest, including the toxicity of treatments, HRQoL or disease recurrence. For these outcomes, which are required for the CEA, we will draw on a review of the published literature. As with any non-randomised study, this study will make assumptions that cannot be tested from the data, but the alternative analyses will assess the robustness of the findings to the key assumptions made in the base case (main) analysis.

### Patient and public involvement

Two public and patient (PP) representatives with lived experience of cancer were involved in the study design from the outset, prior to securing funding and throughout the protocol development process. The PP representatives emphasised the importance of reflecting individuals and their characteristics, such as age, tumour stage, fitness, comorbidities and ethnicity in the study design. The PP representatives and PP study lead convened a PP panel consisting of eight individuals with lived experience of cancer as a patient, carer or community support worker, from diverse backgrounds. The PP panel meets regularly and provides valuable input on key elements of the study, such as the importance of the primary and secondary outcomes and the relevance of subgroups. The PP representatives and panel will continue to assist in identifying the key messages and ensuring that the communication about the study results remains accessible to patients and the general public.

In addition, two clinical experts, a cardiothoracic oncologic surgeon (JE) and a consultant radiation oncologist (CFF), are integral to the study team. These experts contributed to defining the eligibility criteria, treatment definitions, planned analyses and outcome measures and provided feedback on baseline characteristics.

### Deviations

We will publish deviations from the published protocol on the study website https://www.lshtm.ac.uk/research/centres-projects-groups/sort.

## Ethics and dissemination

### Ethics

This study will use data from the national cancer registry, where data are provided by patients and collected by the NHS as part of the routine care (Data sharing agreement: DARS-NIC-656757-J8V9D-v2.3). Patients have the option to opt out of data collection in the cancer registry. Since the collected data does not contain identifiable information, individual consent was not required. Independent ethics approval was obtained by the London School of Hygiene and Tropical Medicine Research Ethics Committee (reference number 29 717–1). The proposed analysis and future interpretation of the results will be carried out and are the responsibility of the authors.

### Dissemination

We will maintain ongoing collaboration with our expert clinical colleagues and PP representatives to share the study outputs and ensure its findings are translated into clinical recommendations for patients with early-stage NSCLC. We will publish the results in open-access journals and present the findings at scientific and clinical conferences. Methodological advancements from this study and future work will be disseminated to facilitate the use of observational evidence in advancing healthcare services and optimising resource utilisation.

## References

[1] Cancer Research UK Twenty most common causes of cancer death. https://www.cancerresearchuk.org/health-professional/cancer-statistics/mortality/common-cancers-compared#heading-Zero.

[2] Exarchakou A, Rachet B, Belot A (2018). Impact of national cancer policies on cancer survival trends and socioeconomic inequalities in England, 1996-2013: population based study. BMJ.

[3] Cancer Research UK Lung cancer survival. https://www.cancerresearchuk.org/health-professional/cancer-statistics/statistics-by-cancer-type/lung-cancer#heading-Two.

[4] National Lung Cancer Audit (2024). State of the nation report 2024: an audit of care received by patients diagnosed with lung cancer in England and Wales during 2022. https://www.lungcanceraudit.org.uk/wp-content/uploads/2024/05/NLCA-State-of-the-Nation-2024_16.05.24_V2.0.pdf.

[5] NICE (2024). Lung cancer: diagnosis and management. https://www.nice.org.uk/guidance/ng122/resources/lung-cancer-diagnosis-and-management-pdf-66141655525573.

[6] Royal College of Radiologists (2020). Radiotherapy for lung cancer RCR consensus statements. https://www.rcr.ac.uk/media/i5spmyvx/rcr-publications_radiotherapy-for-lung-cancer-rcr-consensus-statements_june-2020.pdf.

[7] Ball D, Mai GT, Vinod S (2019). Stereotactic ablative radiotherapy versus standard radiotherapy in stage 1 non-small-cell lung cancer (TROG 09.02 CHISEL): a phase 3, open-label, randomised controlled trial. Lancet Oncol.

[8] Phillips I, Sandhu S, Lüchtenborg M (2019). Stereotactic Ablative Body Radiotherapy Versus Radical Radiotherapy: Comparing Real-World Outcomes in Stage I Lung Cancer. Clin Oncol.

[9] Study details | trial of either surgery or stereotactic radiotherapy for early stage (IA) lung cancer | clinicaltrials.gov. https://clinicaltrials.gov/study/NCT00687986.

[10] Study details | randomized study to compare cyberknife to surgical resection in stage i non-small cell lung cancer | clinicaltrials.gov. https://clinicaltrials.gov/study/NCT00840749.

[11] Franks KN, McParland L, Webster J (2020). SABRTooth: a randomised controlled feasibility study of stereotactic ablative radiotherapy (SABR) with surgery in patients with peripheral stage I nonsmall cell lung cancer considered to be at higher risk of complications from surgical resection. Eur Respir J.

[12] Fernando HC, Timmerman R (2012). American College of Surgeons Oncology Group Z4099/Radiation Therapy Oncology Group 1021: a randomized study of sublobar resection compared with stereotactic body radiotherapy for high-risk stage I non-small cell lung cancer. J Thorac Cardiovasc Surg.

[13] Subramanian MP, Meyers BF (2018). Surgical Resection Versus Stereotactic Body Radiation Therapy for Stage I NSCLC: Can Randomized Trials Provide the Solution?. Cancers (Basel).

[14] Chang JY, Senan S, Paul MA (2015). Stereotactic ablative radiotherapy versus lobectomy for operable stage I non-small-cell lung cancer: a pooled analysis of two randomised trials. Lancet Oncol.

[15] Study details | veterans affairs lung cancer surgery or stereotactic radiotherapy | clinicaltrials.gov. https://clinicaltrials.gov/study/NCT02984761.

[16] Study details | jolt-ca sublobar resection (SR) versus stereotactic ablative radiotherapy (SAbR) for lung cancer | clinicaltrials.gov. https://clinicaltrials.gov/study/NCT02468024.

[17] Senan S (2013). Surgery versus stereotactic radiotherapy for patients with early-stage non-small cell lung cancer: more data from observational studies and growing clinical equipoise. Cancer.

[18] Mokhles S, Verstegen N, Maat A (2015). Comparison of clinical outcome of stage I non-small cell lung cancer treated surgically or with stereotactic radiotherapy: results from propensity score analysis. Lung Cancer (Auckl).

[19] Verstegen NE, Oosterhuis JWA, Palma DA (2013). Stage I-II non-small-cell lung cancer treated using either stereotactic ablative radiotherapy (SABR) or lobectomy by video-assisted thoracoscopic surgery (VATS): outcomes of a propensity score-matched analysis. Ann Oncol.

[20] Matsuo Y, Chen F, Hamaji M (2014). Comparison of long-term survival outcomes between stereotactic body radiotherapy and sublobar resection for stage I non-small-cell lung cancer in patients at high risk for lobectomy: A propensity score matching analysis. Eur J Cancer.

[21] Spencer KL, Kennedy MPT, Lummis KL (2019). Surgery or radiotherapy for stage I lung cancer? An intention-to-treat analysis. Eur Respir J.

[22] Paul S, Lee PC, Mao J (2016). Long term survival with stereotactic ablative radiotherapy (SABR) versus thoracoscopic sublobar lung resection in elderly people: national population based study with propensity matched comparative analysis. BMJ.

[23] Port JL, Parashar B, Osakwe N (2014). A propensity-matched analysis of wedge resection and stereotactic body radiotherapy for early stage lung cancer. Ann Thorac Surg.

[24] Chen H, Laba JM, Boldt RG (2018). Stereotactic Ablative Radiation Therapy Versus Surgery in Early Lung Cancer: A Meta-analysis of Propensity Score Studies. Int J Radiat Oncol.

[25] Ma L, Xiang J (2016). Clinical outcomes of video-assisted thoracic surgery and stereotactic body radiation therapy for early-stage non-small cell lung cancer: A meta-analysis. Thorac Cancer.

[26] Zheng X, Schipper M, Kidwell K (2014). Survival Outcome After Stereotactic Body Radiation Therapy and Surgery for Stage I Non-Small Cell Lung Cancer: A Meta-Analysis. Int J Radiat Oncol.

[27] Li M, Yang X, Chen Y (2017). Stereotactic body radiotherapy or stereotactic ablative radiotherapy versus surgery for patients with T1-3N0M0 non-small cell lung cancer: a systematic review and meta-analysis. Onco Targets Ther.

[28] Maia FH de A, Rozman LM, Carvalho H de A (2023). Systematic review of economic evaluations on stereotactic ablative radiotherapy (SABR) compared to other radiotherapy techniques or surgical procedures for early-stage non-small cell lung cancer. Cost Eff Resour Alloc.

[29] Paix A, Noel G, Falcoz PE (2018). Cost-effectiveness analysis of stereotactic body radiotherapy and surgery for medically operable early stage non small cell lung cancer. Radiother Oncol.

[30] Wolff HB, Alberts L, van der Linden N (2020). Cost-effectiveness of stereotactic body radiation therapy versus video assisted thoracic surgery in medically operable stage I non-small cell lung cancer: A modeling study. Lung Cancer (Auckl).

[31] Puri V, Crabtree TD, Kymes S (2012). A comparison of surgical intervention and stereotactic body radiation therapy for stage I lung cancer in high-risk patients: a decision analysis. J Thorac Cardiovasc Surg.

[32] Louie AV, Rodrigues GB, Palma DA (2014). Measuring the population impact of introducing stereotactic ablative radiotherapy for stage I non-small cell lung cancer in Canada. Oncologist.

[33] Shah A, Hahn SM, Stetson RL (2013). Cost-effectiveness of stereotactic body radiation therapy versus surgical resection for stage I non-small cell lung cancer. Cancer.

[34] Smith BD, Jiang J, Chang JY (2015). Cost-effectiveness of stereotactic radiation, sublobar resection, and lobectomy for early non-small cell lung cancers in older adults. J Geriatr Oncol.

[35] Tataru D, Spencer K, Bates A (2018). Variation in geographical treatment intensity affects survival of non-small cell lung cancer patients in England. Cancer Epidemiol.

[36] Radiotherapy UK Current number of nhs centres in the UK providing advanced radiotherapy with SABR for lung cancer. https://radiotherapy.org.uk/wp-content/uploads/2022/08/RTUK-doc-re-NHS-UK-Centres-providing-SABR-for-lung-cancer.pdf.

[37] Smith BD, Smith GL, Hurria A (2009). Future of cancer incidence in the United States: burdens upon an aging, changing nation. *J Clin Oncol*.

[38] Jørgensen TL, Hallas J, Friis S (2012). Comorbidity in elderly cancer patients in relation to overall and cancer-specific mortality. Br J Cancer.

[39] Spencer K, Jones CM, Girdler R (2021). The impact of the COVID-19 pandemic on radiotherapy services in England, UK: a population-based study. Lancet Oncol.

[40] NHS Digital COVID-19 rapid cancer registration and treatment data dashboard. https://digital.nhs.uk/ndrs/data/data-outputs/cancer-data-hub.

[41] Faivre-Finn C, Fenwick JD, Franks KN (2020). Reduced Fractionation in Lung Cancer Patients Treated with Curative-intent Radiotherapy during the COVID-19 Pandemic. Clin Oncol.

[42] NCRAS National health system digital. https://digital.nhs.uk/ndrs/about/ncras.

[43] Hernán MA, Robins JM (2016). Using Big Data to Emulate a Target Trial When a Randomized Trial Is Not Available. Am J Epidemiol.

[44] Heckman JJ, Urzua S, Vytlacil E (2006). Understanding Instrumental Variables in Models with Essential Heterogeneity. Rev Econ Stat.

[45] Basu A, Heckman JJ, Navarro-Lozano S (2007). Use of instrumental variables in the presence of heterogeneity and self-selection: an application to treatments of breast cancer patients. Health Econ.

[46] Moler-Zapata S, Grieve R, Lugo-Palacios D (2022). Local Instrumental Variable Methods to Address Confounding and Heterogeneity when Using Electronic Health Records: An Application to Emergency Surgery. Med Decis Making.

[47] Hernán MA, Wang W, Leaf DE (2022). Target Trial Emulation: A Framework for Causal Inference From Observational Data. JAMA.

[48] Henson KE, Elliss-Brookes L, Coupland VH (2020). Data Resource Profile: National Cancer Registration Dataset in England. Int J Epidemiol.

[49] Chang JY, Mehran RJ, Feng L (2021). Stereotactic ablative radiotherapy for operable stage I non-small-cell lung cancer (revised STARS): long-term results of a single-arm, prospective trial with prespecified comparison to surgery. Lancet Oncol.

[50] Bidulka P, Lugo-Palacios DG, Carroll O (2024). Comparative effectiveness of second line oral antidiabetic treatments among people with type 2 diabetes mellitus: emulation of a target trial using routinely collected health data. BMJ.

[51] Oken MM, Creech RH, Tormey DC (1982). Toxicity and response criteria of the Eastern Cooperative Oncology Group. Am J Clin Oncol.

[52] Smith T, Noble M, Noble S (2015). The English indices of deprivation 2015 technical report. https://assets.publishing.service.gov.uk/media/5a7f24b240f0b62305b85578/English_Indices_of_Deprivation_2015_-_Technical-Report.pdf.

[53] Tyczynski J, Démaret E, Parkin D Standards and guidelines for cancer registration in Europe. https://publications.iarc.fr/_publications/media/download/7000/e30c95c74287c49b08334eaedd3aa12ef31e7e81.pdf.

[54] Royston P, Parmar MKB (2002). Flexible parametric proportional-hazards and proportional-odds models for censored survival data, with application to prognostic modelling and estimation of treatment effects. Stat Med.

[55] Charvat H, Remontet L, Bossard N (2016). A multilevel excess hazard model to estimate net survival on hierarchical data allowing for non-linear and non-proportional effects of covariates. Stat Med.

[56] Pohar Perme M, Estève J, Rachet B (2016). Analysing population-based cancer survival - settling the controversies. BMC Cancer.

[57] Bartlett JW, Harel O, Carpenter JR (2015). Asymptotically Unbiased Estimation of Exposure Odds Ratios in Complete Records Logistic Regression. Am J Epidemiol.

[58] Baiocchi M, Cheng J, Small DS (2014). Instrumental variable methods for causal inference: Instrumental variable methods for causal inference. Stat Med.

[59] Blom EF, Haaf KT, de Koning HJ (2020). Systematic Review and Meta-Analysis of Community- and Choice-Based Health State Utility Values for Lung Cancer. Pharmacoeconomics.

[60] Jovanoski N, Abogunrin S, Di Maio D (2023). Health State Utility Values in Early-Stage Non-small Cell Lung Cancer: A Systematic Literature Review. Pharmacoecon Open.

[61] Wolff HB, Alberts L, Kastelijn EA (2018). Differences in Longitudinal Health Utility between Stereotactic Body Radiation Therapy and Surgery in Stage I Non-Small Cell Lung Cancer. J Thorac Oncol.

[62] Stinnett AA, Mullahy J (1998). Net health benefits: a new framework for the analysis of uncertainty in cost-effectiveness analysis. Med Decis Making.

[63] NHS (2025). National cost collection 2023/24. https://www.england.nhs.uk/costing-in-the-nhs/national-cost-collection/.

[64] Jones, Burns (2021). Costs of health and social care 2021. https://www.pssru.ac.uk/project-pages/unit-costs/unit-costs-of-health-and-social-care-2021/.

[65] NIHR Funding and Awards Identifying cancer recurrence within patient care pathways across linked national clinical datasets. https://fundingawards.nihr.ac.uk/award/NIHR132459.

[66] Teke ME, Sarvestani AL, Hernandez JM (2022). A Randomized, Phase III Study of Sublobar Resection (SR) Versus Stereotactic Ablative Radiotherapy (SAbR) in High-Risk Patients with Stage I Non-Small Cell Lung Cancer (NSCLC). Ann Surg Oncol.

[67] Manca A, Hawkins N, Sculpher MJ (2005). Estimating mean QALYs in trial-based cost-effectiveness analysis: the importance of controlling for baseline utility. Health Econ.

[68] Khakwani A, Rich AL, Powell HA (2015). The impact of the “hub and spoke” model of care for lung cancer and equitable access to surgery. Thorax.

[69] Staiger D, Stock JH (1997). Instrumental Variables Regression with Weak Instruments. Econometrica.

[70] Montiel Olea JL, Pfleuger C (2013). A Robust Test for Weak Instruments. J Bus Econ Stat.

[71] Moler-Zapata S, Grieve R, Basu A (2023). How does a local instrumental variable method perform across settings with instruments of differing strengths? A simulation study and an evaluation of emergency surgery. Health Econ.

[72] Basu A, Coe NB, Chapman CG (2018). 2SLS versus 2SRI: Appropriate methods for rare outcomes and/or rare exposures. Health Econ.

[73] Schafer JL, Kang J (2008). Average causal effects from nonrandomized studies: a practical guide and simulated example. Psychol Methods.

[74] Rutherford MJ (2020). NICE dsu technical support document 21. Flexible methods for survival analysis. https://nicedsu.sites.sheffield.ac.uk/tsds/flexible-methods-for-survival-analysis-tsd.

